# Screening of caspase-3 inhibitors from natural molecule database using e-pharmacophore and docking studies

**DOI:** 10.6026/97320630015240

**Published:** 2019-03-31

**Authors:** Sasidhar Reddy Eda, Ganesh Kumar Veeramachaneni, Jayakumar Singh Bondili, Rajeswari Jinka

**Affiliations:** 1 Department of Biochemistry, Acharya Nagarjuna University, Guntur, Andhra Pradesh, India; 2 Department of Biotechnology, K L E F,Green Fields, Vaddeswaram, Guntur Dist, India

**Keywords:** Caspase 3, Screening, Docking studies, MM-GBSA

## Abstract

Caspase a protease family member, have a vital role in cell death and inflammation process. Caspase-3, an effector caspase controls the regulation of apoptosis and has an anti apoptotic function. The mechanical significance of restoring apoptosis signaling to selectively target malignant cells is utilized to develop strong therapeutic strategies by the caspase family of mortality - induction molecules. Caspase-3 has currently no clear role in treatment for tumor progression and tumor sensitivity. The present study was aimed to screen caspase for potential inhibitors using computer aided docking methodologies. For this, zinc natural molecule database molecules were screened using e-pharmacophore and ADME protocols along with docking studies. Docking analysis selected two molecules, namely ZINC13341044 and ZINC13507846 with G-scores -5.27 and -6.19 respectively. These two potential hits are predicted as caspase inhibitors based on the results and can be further processed for in vitro validation.

## Background

The Extracellular matrix (ECM) receptors are imperative controllers of angiogenesis. One of these receptors, integrin α5β1, impact tumor-cell survival, multiplication, and metastasis, since the adversaries of this integrin α5β1 strongly restrain angiogenesis and tumor development 1. Unligated α5β1 integrin inhibits survival and proliferation of the tumor cell even when they adhere to the ECM through different integrins assuming a major role in the direction of cell survival 2, 3. On the other hand, for certain biochemical and morphological changes during apoptosis, Caspase- 3 is required. It is a frequently activated death protease, which cleaves a range of important cell proteins with numerous death signals. This is also important for cell death in a significant manner based on tissue, cell - type or death stimulus, as it is essential for the implementation and completion of apoptosis in certain types of characteristic cell morphology changes and biochemistry events. However, the specific requirements of this caspase in apoptosis were largely unknown 4. Few reports show that integrin and caspases interact directly, although caspases were activated via integrin generated signaling pathways 5. In the plasma membrane of the rat fibroblast cells during late stages of anoikis, our previous data reported the direct interaction between α5β1 integrin and caspase 3. These cells avoid cell death through the interaction of caspase 3 and unligated α5β1 integrins during the non - adherence process 2. Screening of natural molecules for their biological activity using in vitro protocols is a time-consuming process and success ratio was also low. In silico methods became prominent in screening of lead molecules by reducing experimental time and eliminating false positives. The aim of the present study was focused mainly on screening of small, potent inhibitors against caspase-3 protein.

## Methodology

### Protein preparation and grid generation:

The 3D crystalized structure of caspase protein (PDB ID: 5IBC) was retrieved from the protein data bank 6. The protein was prepared by using the protein prep wizard 7, helps in converting the raw structure to a refined structure. Major steps in the preparation involve addition of hydrogen, removal of unwanted water molecules beyond 5 Å, optimizing and minimizing the structure. The active pocket in the prepared protein was freezed by using the receptor grid generation.

### Database preparation:

Zinc natural molecules database 8 were retrieved, conversion of molecules structure from 2D to 3D and refinement steps were carried using the canvas module from the Schrodinger software 9, 10. Further through Conf-Gen application 11 the molecules confirmations were generated.

### Pharmacophore hypothesis generation and database screening:

Two methodologies, structure-based drug-design and ligand-based drug-design are renowned important in silico screening approaches in drug discovery pipeline. e-Pharmacophore based methodology combines both structure-based and ligand-based methods to screen the molecule database 12, 13. Using the crystal protein-ligand complex a hypothesis was generated and further screened the database by setting the application values to default. Further the screened molecules were subjected to QikProp for ADME analysis 14.

### Docking studies:

The screened molecules were docked into the active site of the caspase protein with the help of XP docking protocol 15-17 of the glide application. The application run was carried by choosing the protein grid file, screened molecules, setting docking protocol to XP and remaining options to default. The complexes were evaluated based on the binding modes between the protein and ligand along with the G-scores. The G-scores were calculated based on the following formula 

Glide score =0.065 * vdW +0.130 * Coul + Lipo + Hbond + Metal + BuryP + RotB + Site

vdW - van der Waals energy, Coul - Coulomb energy, Lipo represents lipophilic term derived from hydrophobic grid potential, Hbond - hydrogen-bond, Metal - metal-binding term, BuryP - buried polar groups, RotB - penalty for freezing rotatable bonds, and Site - polar interactions in the active site.

### Prime/MM-GBSA

Binding free energies of the final complexes were calculated using Prime/MM-GBSA 18 in the presence of OPLS force field 19, 20 in VSGB solvent model.

## Results and Discussion

Initially, for the generation of pharmacophore hypothesis the crystal protein was separated into protein and ligand. With the receptor grid generation application, a grid was generated around the active site and the crystal ligand was docked into that grid boxed active site using Glide XP protocol. The ligand orientation after docking was cross verified with crystal structure orientation, same orientation was reproduced confirming the docking protocol is valid and further used for docking studies.

### Screening studies:

**Figure 1:**
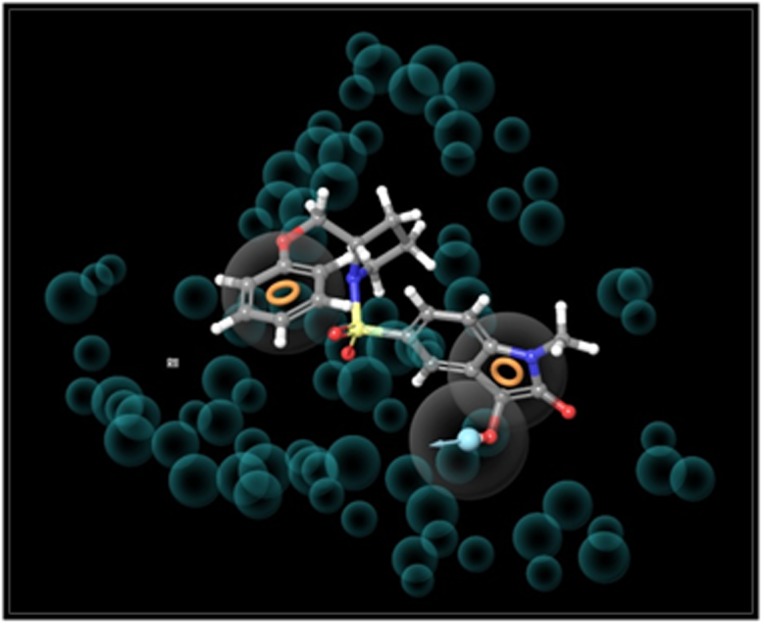
RRD (three sites) hypothesis generated using the e- Pharmacophore application

e-pharmacophore based screening was carried using the re-docked pose viewer file, a three site hypothesis RRD (R represents ring and D represents donor) was generated (Figure 1). By setting all the parameters to default in the Phase screening protocol, the molecules database was screened with the hypothesis and a total of 32 hits were retrieved based on 3 out 3 matching criteria, i.e. all the three features of crystal molecule must be full filled by the screened molecule. Matched hits were further screened using QikProp application by choosing CNS, human oral absorption and Lipinski's rule of five as the major criteria's (Table 1). Out of those 32 molecules, 7 molecules passed CNS as -2 (Predicted inactive against central nervous system), percent human oral absorption in between 80 to 100 and Lipinski's rule violations as zero (rule of five includes mol_MW <500, QPlog Po/w <5, donor HB = 5, accpt HB = 10 and the compounds which fulfill these rules were considered as druglike).

### Docking studies:

The seven hits attained from the screening studies were subjected to molecular docking studies. Using Glide XP docking protocol, the molecules binding affinity with the amino acids present in the active pocket of the protein were studied.

Binding Interactions of Caspase-3 with the screened molecules ZINC06036807 molecule made five back-bone hydrogen bonds and one pi-pi interactions with the active pocket amino acids. Amino acids involved in the hydrogen bond formation are His 121 of chain A and Arg 207, Trp 206, Phe 250 and Ser 251 of chain b with the molecule.one pi-pi interaction was observed between molecule and Trp 206 residue of chain B. Three hydrogen bonds were observed between the active pocket of caspase-3 and ZINC13507846 by the end of docking studies. All the hydrogen bonds were maintained with the chain B residues, two hydrogen bonds with Ser 205 and the remaining hydrogen bond was observed with Ser 209 by the molecule. Apart from the hydrogen bond, no other interactions were observed in this complex. Binding affinity between active pocket amino acids of caspase-3 and ZINC01642250 was maintained with two hydrogen bonds and one pi-pi interactions. Chain B residues Ser 205 and Trp 206 made back bone hydrogen bonding with ZINC01642250 and one pi- pi interaction was between Trp 206 and ZINC01642250.

A total of five interactions were observed between caspase -3 active pocket and ZINC00056474. Out of five interactions, four are back chain hydrogen bonds and the remaining was a pi-pi interaction. All the interactions were observed with the chain B residues, Arg 207, Ser 205 residues made two hydrogen bonds individually and Trp 206 made pi-pi interaction with the ligand molecule. ZINC31167269 molecule produced four back bone hydrogen bonds by the end of docking studies. Three hydrogen bonds were with the chain B residues and one with the chain A residue present in the binding site of the protein. Chain B residues Arg 207 produced two hydrogen bonds and Ser 205 made one hydrogen bond with ZINC31167269. One residue from the chain A, His 121 made hydrogen with the ligand. ZINC13341044 and caspase-3 complex was maintained through three back bone hydrogen bonds and three pi-pi stacking�s. Two amino acids from chain A, Gly 122 and His 121 are involved in the hydrogen bond formation with the ligand molecule. Chain B residues Arg 207 produced one hydrogen bond and three pi - pi stacking's by the residues Trp 206, Tyr 204 and Phe 256 with the ZINC13341044 molecule. Last molecule ZINC13507842 made two back bone hydrogen bonds with the Arg 207 and Ser 205 of chain B. Prime based MM-GBSA energy calculation was carried to the seven complexes and their energies were tabled in the Table 1 along with their G-scores.

**Figure 2:**
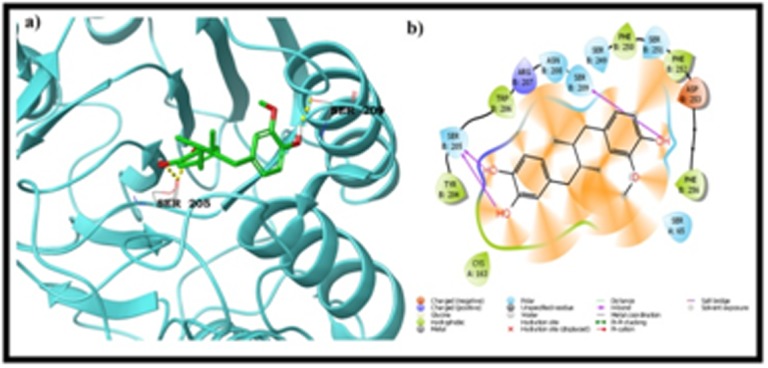
Binding mode of Zinc1341044 with caspase 3 a) 3D view b) LigPlot

**Figure 3:**
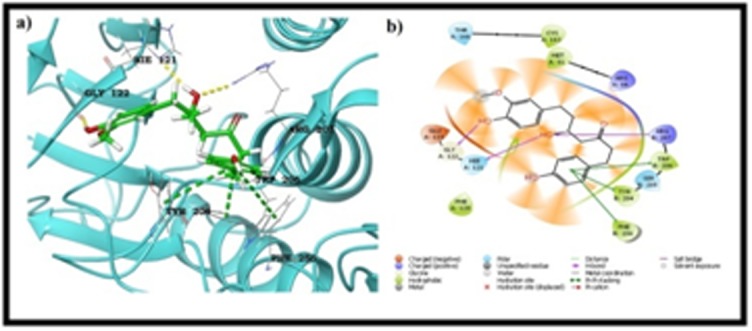
Binding mode of Zinc13507846 with caspase 3 a) 3D view b) LigPlot

**Figure 4:**
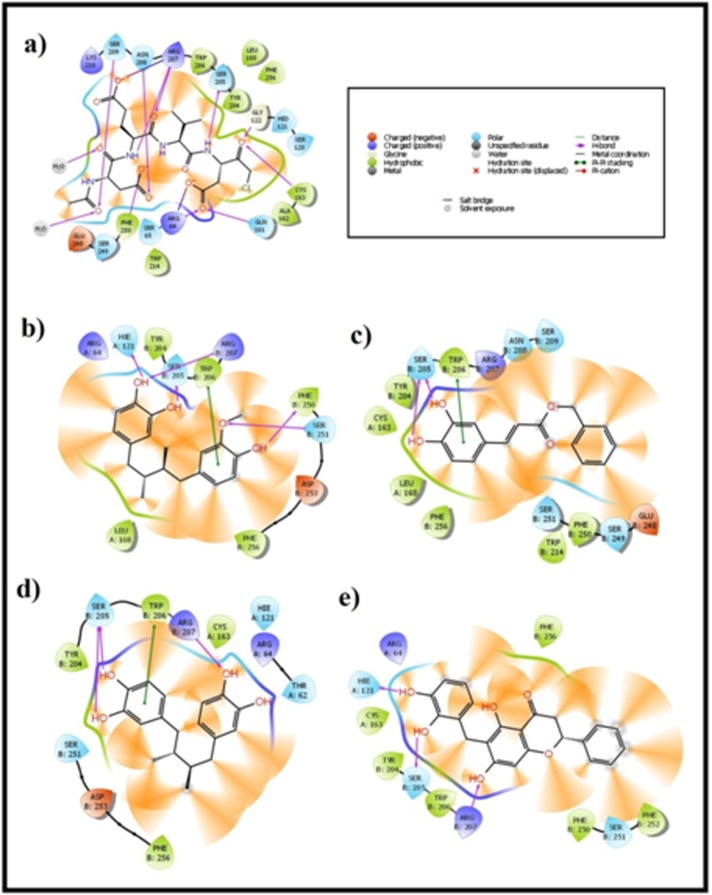
Ligand interaction diagram of (a) Crystal ligand from pdb structure (ID: 5IBC) (b) ZINC06036807 (c) ZINC01642250 (d) ZINC00056474 and (e) ZINC31167269

From the previous studies, it was evident that Tyr 204, Ser 205, Ser 209 and Ser 251 of chain B are very crucial amino acids in the active site of the caspase 3 for activity 21. Apart from them Gly 122 form chain A also plays an important role in inhibiting the caspase 3 activity 22, 23. The present study is projected on screening the natural molecule database targeting them as potential hits as caspase 3 inhibitors. The end of screening protocols reported a total of seven molecules and these molecules were docked into active pocket of the protein. All the seven molecules fitted well inside the pocket and produced interactions with the amino acids present in the binding region. Among seven hits, one hit i.e. ZINC13341044 produced interactions with important amino acids, i.e. Gly 122 (Hbond), Tyr 204 (π - π) and Trp 206 (π - π), similar to that of the crystal ligand. The interaction profile of protein - ZINC13341044 was depicted in Figure 2. Remaining other molecules failed to produce interaction with Gly 122, but they produced interactions with the other important amino acids like Ser205 and Ser 251. ZINC13507846 molecule produced important hydrogen bonds with important residues Ser 205 and Ser 209, which was considered as the second best hit from the binding studies (Figure 3). These two molecules were reported as best molecules based on the H-bond formation with the important amino acids in the active site, Gscores and energy. Binding poses of Crystal ligand (id: 5IBC), ZINC06036807, ZINC01642250, ZINC00056474 and ZINC31167269 were represented in Figure 4a-e.

## Conclusion

The main objective of our present study is to screen natural molecules database to select small molecules as inhibitors against caspase-3. Analysis selected two potential hits against the target namely ZINC13341044 and ZINC13507846 based on the binding mode with the target active site amino acids, satisfying all the ADME important descriptors and with good energy calculations. The caspase-3 inhibition activity for these molecules can be validated using in vitro and in vivo methods.

## Conflict of Interest

Authors declare no conflict of interest.

## Figures and Tables

**Table 1:** List of molecules with ADME, glide scores and energies

Molecule	ADME			Glide	Prime Energy
	CNS	Percent Human	Rule Of Five	G-score	MM-GBSA
		Oral Absorption			
ZINC06036807	-2	93.39	0	-6.78	-35.03
ZINC13507846	-2	93.85	0	-6.19	-42.9
ZINC01642250	-2	82.74	0	-6.19	-51.84
ZINC00056474	-2	85.08	0	-6.04	-40.84
ZINC31167269	-2	88.37	0	-6.56	-32.94
ZINC13341044	-2	86.34	0	-5.27	-42
ZINC13507842	-2	92.7	0	-4.98	-32.9
